# The Impact of Retired Immigrants on Quality of Life for the Local Aging Population: Results from the Southeast Spanish Coast

**DOI:** 10.3390/ijerph20010366

**Published:** 2022-12-26

**Authors:** José Ramón Valero-Escandell, Xavier Amat-Montesinos, Carlos Cortés-Samper

**Affiliations:** Department of Human Geography, University of Alicante, Carretera San Vicente del Raspeig s/n, 03690 San Vicente del Raspeig, Spain

**Keywords:** residentialism, residential environments, elderly migrations

## Abstract

The immigration of foreign retirees to Spain’s southeast coast is a tradition that stretches back decades. This phenomenon has modified the demographic structure of many towns and also transformed their social and economic features, as well as the number and diversity of services available to the population. This study examines the effect of post-employment immigration on economic revitalisation, the increase in services and the arrival of new inhabitants. It demonstrates the transformative potential of the phenomenon for pre-coastal areas in the Spanish southeast and its influence on the configuration of friendly residential environments. The study analyses the evolution of basic demographic data and its spatial behaviour, establishing relationships between the quality of life of the local aging population and retired immigrants. The analysis focuses on the impact of residentialist areas on the configuration of friendly living environments. The results show how these communities, which were once somewhat stagnant and had a significantly aging population, either participated in the development or have improved their access to certain services and facilities, configuring new environments. The results reflect the improvement of the quality of life for the elderly in these settings, considering that they are often the majority age group.

## 1. Introduction

Currently, foreign retirees are immigrating in significant numbers all around the world [1,2,3,4], with one of the most affected areas being the Spanish Mediterranean coast. This movement has led to a phenomenon known as residentialism [5], or more commonly, residential tourism [6,7], in the communities that receive these populations.

Although the concept of residential tourism may be useful in political discourse on urban transformation or to refer to the tourist base that produces the phenomenon, the term is not truly accurate: the individuals in these residential groups no longer qualify as tourists, as they generally reside in the location. This, technically, makes them immigrants, albeit with special characteristics (they are not workers, they arrive at an advanced age and often return to their place of origin). Moreover, residentialism is directly linked to active ageing. It represents a challenge for both the destinations and the members of the group who have changed their living environment late in life. The concept is similar to the lifestyle migration described by Benson and O’Reilly [8].

Lifestyle migrations and residentialist processes are common to much of the European Mediterranean region [9]. A number of previous studies have analysed it in depth, focusing on British communities established in areas like Tuscany, Malta and the Costa del Sol [10] that have formed what has come to be called ‘Little England’ [11]. Other authors have included French locations far from the Mediterranean in their research [12]. In any event, all the authors generally agree that residentialism is linked to the prior development of tourism in these areas.

Southeast Spain is a major enclave of residentialism in Europe [13]. The group began to grow slowly with the advent of mass tourism in the area of Benidorm (Alicante) [14] and extended to different beach areas between the cities of Valencia and Almeria [15]. In 1976, the sociologist Mario Gaviria [16] published a book on winter tourism and settlements of foreigners in Alicante. The publication raised awareness about a phenomenon that concerned thousands of residents, despite being highly localised. In the 1986 regional population census [17], the process had visibly taken root in La Nucia and Alfaz del Pi, two towns in the Marina region of Benidorm, whose foreign populations at the time represented 59.1% and 52.4% of the total, respectively. The elderly, in particular, accounted for 37.4% of the foreigners in Alfaz del Pi and 36.2% in La Nucia. Additionally, the concentration of certain national communities, such as Norwegians, was notably heavy in these municipalities [18]. The situation in the nearby town of Calpe was similar, though less extreme. All these small towns saw their populations double in 15 years, with more than 3000 resident foreigners and an activity rate significantly lower than the provincial average. In the province of Almeria to the south, the phenomenon had also begun in the tourist area of Mojácar, although it had not yet reached the same levels as in Alicante [19].

The premise of this study is that residentialist environments are not immutable. They evolve, grow and also shrink over time. Despite this instability, residentialist areas possess the characteristics defined back in 1979 by Jurdao [20] in the case of the Costa del Sol town of Mijas (Malaga). The population almost always lives in new urban centres that are not often linked to the traditional consolidated centre and are sometimes only connected via administrative dependence on that municipality. Most residents are foreigners and homeowners, whose actual place of residence changes throughout the year. Their income is higher than that of local residents in the same situation, in the form of higher pensions. Finally, they do not integrate easily and frequently engage in property transactions.

In some cases, communities that are almost exclusively national have emerged, and they are cut off from the traditional society in the area, even in spatial terms [21]. This type of peripheral urbanisation is linked to the interesting and politicised concept of residential tourism, which is commonly associated with simple tourism and financial concerns [22]. Residentialism can bring about excessive real estate development [23], generating visible socio-spatial fragmentation [24] and a marked dualism in each area’s demographic structure. Some authors have argued that residentialism is an unsustainable territorial process [25], while others emphasise territorial spreading patterns based on specific expansion processes [13]. The phenomenon has often attracted critical analysis that highlights the ecological and sociocultural costs. However, it also has its supporters, who compare it to tourism and the need for an economic alternative based on real-estate-related construction [26].

Residentialism reached its peak in the province of Alicante in the early 2010s. Using statistical data, we compared the evolution of residentialist towns with other municipalities between 2001 and 2021, focusing on the transformation of the municipalities that reached residentialist status, as defined below, in the early 2000s.

Based on the above, the study objectives were as follows:

1. To interpret the demographic evolution and aging process of residentialist towns on Spain’s southeast coast.
2. To characterise the impact of retirement colony settlements on various social and demographic aspects.
3. To explore the relationship between residentialism and the local older native population.
4. To identify the connection between residentialism and urban planning at a local level, particularly in relation to the configuration of friendly environments.

## 2. Materials and Methods

### 2.1. Bibliographical Review

The literature review focused both on the settlement of retired foreign migrant communities in the territory analysed—southeast Spain and especially the province of Alicante because of its singular spatial concentration—and on the quality of life for the elderly there, along with the territorial transformations that occurred in the areas where foreigners settled.

The bibliography on the relationships between tourism development and the emergence of residentialism was reviewed, with an emphasis on the transition between the repeat tourist and the stable immigrant who has chosen to settle. Additionally, references to spatial transformations were consulted, especially those related to urban planning and the rapid growth of municipalities. Likewise, the investigation identified references that link this type of migration to the quality of life of the elderly.

Following this analysis, a number of variables were established according to the available statistical data.

### 2.2. Statistical Analysis

We began by determining the criteria that characterises a residentialist population. The elements considered were as follows:

1. Over 25% of the population are foreigners. This percentage is much higher than the national average and illustrates the massive presence of a non-native population. It denotes a notable demographic change in a country where foreign immigration was rather rare until the mid-1980s. However, it is not a sufficient condition by itself because some towns have similar percentages that are due to labour immigration.
2. Over 66.6% of the foreigners are European, in most cases from North Atlantic countries. This percentage reached 90% or more in almost all the towns studied. Where labour immigration predominates, the main groups tend to be from Africa or Latin America.
3. The average population age in the municipality exceeds the average of the total provincial population by over two years. In some non-residentialist cities, many European foreigners are attracted by job opportunities (e.g., Romanians and Bulgarians). However, in these cases, young adults predominate. A high average age is more strongly associated with the settlement of retired immigrants than with the working population, since the latter, arriving mainly between 1990 and 2010, still does not have a high percentage of elderly people (not even amongst Romanian groups).

A municipality that met all three of these conditions was characterised as residentialist, as there is a large foreign group, but its reasons for immigrating are not work-related. However, if the foreign population accounts for between 15 and 25% of the municipal population, it is considered a semi-residentialist town, as long as it meets the other two criteria.

This characterisation was based on municipal statistical information available from the National Statistics Institute of Spain [27]. The main sources were the Continuous Register Statistic [28] and the household income distribution map (experimental statistics) [29], which made it possible to calculate the total population, the percentage of foreign residents in each location, the percentage of Europeans in this group, household size, single people, and average income. We also used the list of health and care establishments issued in 2020 by the Ministry of Health of the Generalitat Valenciana [30], which identifies private businesses, including opticians, orthopaedic clinics and dental clinics. With these data, it was possible to establish correlations with the percentage of European foreigners in any given year.

### 2.3. Spatial Analysis

The statistical values were represented spatially in order to interpret the territorial distribution of the residentialist towns and the spatial evolution of this settlement process, which in almost all cases is related to an increase in the immigrant population.

According to official 2001 population data [31], 18 municipalities in the province of Alicante could be considered residentialist. Together, they accounted for 171,882 inhabitants, i.e., 11.5% of the total population in the province. In 10 municipalities, foreigners represented over 40% of the residents. In addition, nine municipalities could be considered semi-residentialist, having a foreign population of 15–25% and meeting the other two conditions. To verify the differences, we compared their evolution to that of the rest of the province up to 2021. In 2001, no appreciable residentialist trend could be found in the neighbouring provinces of Murcia and Valencia, where settled foreign retirees did not constitute a notable social phenomenon. In Almeria, only two municipalities were in a similar situation: Mojácar and, to a much lesser extent, Bédar. For this reason, given its specific characteristics and the existence of earlier related studies [32], the analysis focused on the province of Alicante.

To support the spatial analysis, we also consulted the municipal urban planning documents of the 38 towns that achieved residentialist status between 2001 and 2021. This analysis was based on the information available from the database of the Ministry of Territorial Policy, Public Works and Mobility [33] created from the urban planning documents in force in 2022. The data obtained explained how residential areas were distributed and evolved spatially. It also made it possible to determine the planning and location of age-friendly public urban services targeting the residentialist group.

## 3. Results

### 3.1. Evolution of the Population

Table 1 illustrates the evolution of the population of the province of Alicante, the most clearly residentialist group on Spain’s southeast coast, from 2000 to 2020.

The data reveal a number of trends:

1. The population rose significantly more during these two decades in residentialist towns than in the province as a whole. This was particularly visible in the small, semi-residentialist towns, many of which are now becoming established destinations. Some municipalities saw their European groups grow more, such as San Fulgencio and Rojales (Vega Baja), whose populations doubled.
2. Despite the above, the evolution of the population was more unstable over time. The population grew rapidly in the 2000s, but then slowed significantly, largely because of a drop in European residents and in the elderly population in particular. This instability can be explained by a wide range of factors: the consequences of the post-2008 financial crisis; the impact of Brexit on the perceptions of the British—the largest established group in Alicante; the 2020–2021 global pandemic, which led many people to return to their places of origin; and additional complementary factors, such as the Spanish treasury’s tighter control on the foreign wealth of residents in the country, which may discourage individuals from registering as Spanish residents.
3. The average age of residentialist town inhabitants, which was very high because of the mass settlement of elderly people, continued to increase. However, in both residentialist and semi-residentialist communities, the average age increased less than in the province of Alicante as a whole. Spain’s rapidly ageing population—due more to extremely low birth rates than to increasing life expectancy—has caused the average age in the province to rise by 4.1 years in just 20 years. Although it is still lower than that of the residentialist municipalities, the gap has narrowed. Moreover, a number of the most emblematic residentialist municipalities have ceased to meet residentialist criteria because their average ages are very similar to the provincial average. In some cases, it is even lower, such as in La Nucia, the first municipality to have a foreign majority, in 1986.

### 3.2. The Spatial Advance of Residentialism

**Figure 1:**
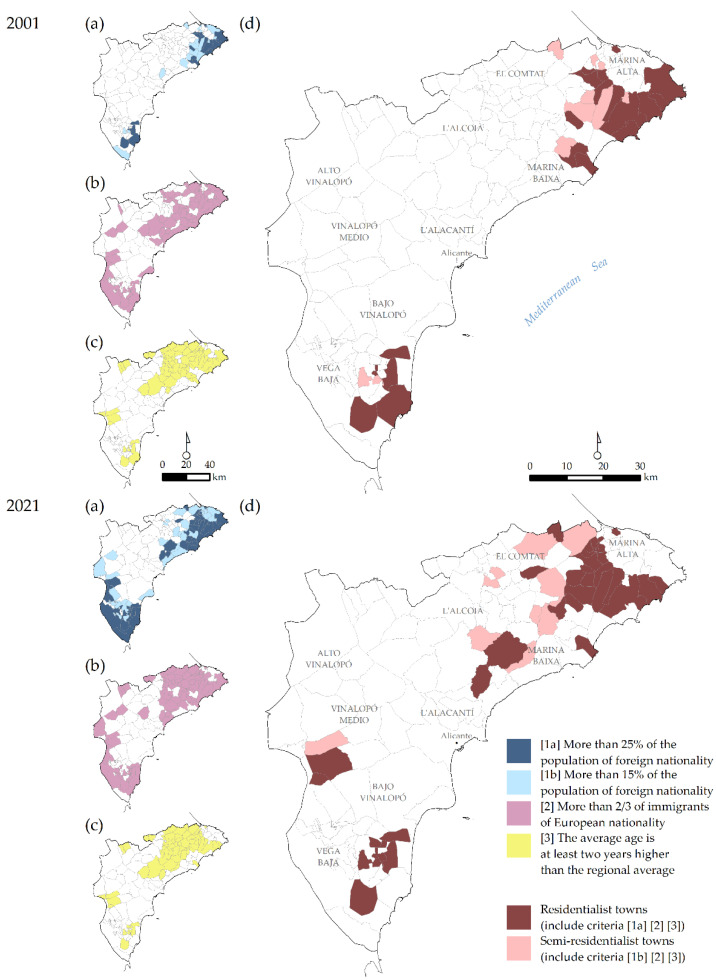
Distribution of residentialist municipalities in 2001 and 2021. (**a**) Municipalities with more than 15 or 25% of the population of foreign nationality. (**b**) Municipalities with more than 2/3 of immigrants of European nationality. (**c**) Municipalities where the median ages are at least two years higher than the regional median age. (**d**) Residential or semi-residential towns according to criteria in (**a**–**c**).

Spatially, residentialist areas transformed substantially during the period studied. Figure 1 shows the residentialist municipalities based on 2001 and 2021 data. In 2001, the impact was highly localised in two specific areas: (a) the coast and pre-coastal areas of the Marina Alta and Baixa districts, north of Benidorm and south of Denia, forming a distinct continuity between Calpe and Jávea; and (b) a smaller territory around Torrevieja, concentrated in two pre-coastal municipalities—San Fulgencio and Rojales—characterised by large urbanisations of foreign residents. Almost no residentialist municipalities were found outside this area.

By 2021, residentialism had clearly cut its direct link to the coast, where it only remains in three coastal municipalities, none of which are amongst those with the largest population. The number of residentialist towns in the province, however, has increased. Residentialism is located in the pre-coastal areas, essentially where it began (Marina Alta and Baixa and Vega Baja, northern and southern province). It has also extended to other towns, almost all of which are rural, and in some cases have a small number of inhabitants. These areas, l’Alacantí and Vinalopó, have traditionally been unaffected by residentialism and tourism itself, and include the towns with the poorest connections in the inland mountains.

### 3.3. Sociodemographic Trends

Table 2 shows various types of correlations for all the municipalities in the province of Alicante between the key defining factor of residentialism—the weight of European residents—and other sociodemographic and economic factors, including the potential link between the share of European foreigners in each location and average household size, the percentage of single-person households, average income per person, or the available health and care services.

There is a notable positive relationship between the percentage of Europeans in a municipality and its population growth. There is also a somewhat minor, inverse relationship with respect to the average age evolution, a process completely different to the initial decades of the phenomenon. The data confirm the previous results, although—notably—no major differences regarding the total for the province were found, including when only rural and semi-rural municipalities in Spain with fewer than 10,000 inhabitants are considered.

The household income distribution map provides detailed data at local—and even district—level on the structure of the household between 2015 and 2019. Both years present a remarkable inverse correlation between the household size and the population of European origin. It is not difficult to advance a plausible hypothesis: the percentage of the elderly population was very high amongst European residents, i.e., immigrants who arrived in their retirement years, which can often be relatively early. This population usually settles down in couples, and for age reasons, almost never have children or parents in their care—factors that would, additionally, hinder a change in residence. With few large families, and even few two-generation families, household sizes cannot be large.

Over these same years, hardly any correlation could be found between the percentage of single-person households, single people and the settlement of Europeans in certain municipalities. The relationship varied depending on the rural factor, although the values remained low. Today, Spanish society is aging, and the average population age is higher in many rural, non-residentialist municipalities than in communities with a high percentage of foreign retirees.

Less logical, at first sight, is the negative correlation between the income level and the percentage of settled Europeans. Much of the European population originally arrived—and is still arriving—because of the more affordable prices in Spain than in their countries of origin that are due to the significantly lower income than that of some North Atlantic countries. It is difficult to explain this correlation, as it seems unlikely that European settlers in Spain are poorer than the native population.

Significantly, the income data comes from the tax information kept by the Spanish Treasury, which influences this factor. A large portion of the elderly immigrant population in Spain depends on the social security pension from their country of origin, which is usually higher than Spanish pensions [34]. Moreover, many of their pensions are exempt from income tax, which is due both to the characteristics of these pensions and because of agreements that prevent double taxation. A large number of immigrants who continue to maintain some type of economic activity in their country of origin do not usually register their actual residence, even if they stay in Spain for long periods of time. Consequently, tax information is not a suitable means to characterise this group in economic terms.

Finally, each municipality’s population was correlated with the presence of three types of health establishments: opticians, orthopaedic clinics and dental clinics. These are all private, since the public medical infrastructure (e.g., health clinics) is usually evenly distributed across the province, according to each municipality’s population size. The aim was to determine whether the increase in the elderly population of European origin leads to a greater likelihood of these types of businesses in their municipalities of residence. Not only did this not appear to be the case, but there even seemed to be a slight inverse correlation. As this immigration group would be the logical target client for such establishments, the lack of a customer base cannot be the reason. The explanation may be, rather, that district capitals monopolise most of these businesses within their own spheres of influence, and that is not where residential groups tend to concentrate.

In short, the only clear trend found is a population increase—beyond the demographic size potential—in the areas of settlement and the relationship with smaller average households. The other correlations were not significant. Residentialism today appears to be much more scattered across the province of Alicante than it initially was. Moreover, foreign retirees contribute only partially to the striking aging process that characterises Alicante today. Yet the group differs from natives in a number of ways: they predominate in different places; they have problems integrating into the host society (language being the major, but not only, reason); their settlement is easily reversible in the event of loss of personal autonomy or death of the partner; and the care required is much more complex. For multiple reasons, therefore, creating age-friendly environments for them is a challenge—and not only in terms of building material infrastructure.

### 3.4. The Transformation of Residential Areas

**Figure 2:**
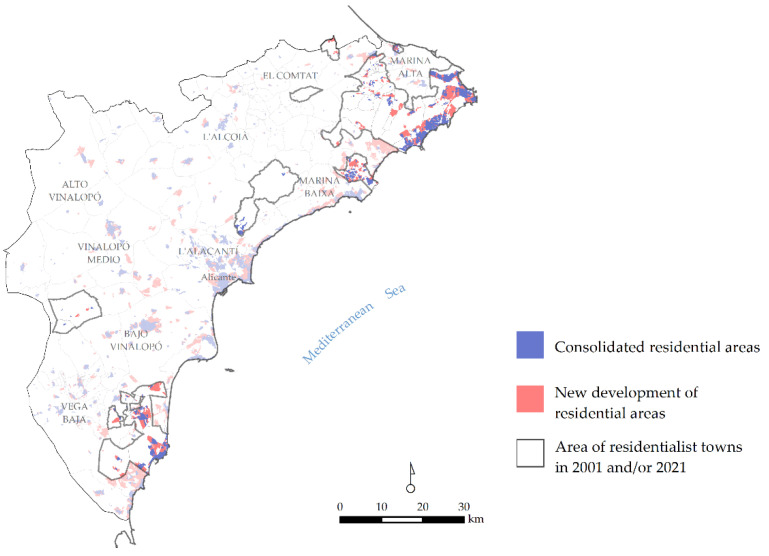
Urban growth in residentialist localities according to urban planning.

Residentialism has bloated the population of some towns and boosted the local economy across different sectors, from construction to services. The process has not always been similar in all communities, nor has it evolved at the same pace. Some towns have seen tremendous population growth, and others a more moderate rise. In all cases, urban planning has decisively influenced the generation of residentialist locations through the design of new areas for the newly arrived inhabitants. As seen in Figure 2, in most cases, new residentialist areas have spread beyond the traditional town limits, producing unprecedented urban arrangements with a variety of problems and needs.

Urbanisation processes have largely been linked to residentialism, and vice-versa [35]. Residentialist communities are primarily located in areas with the largest amount of space suitable for new housing. During the first stage of residentialism, urban expansion was accompanied by a degree of densification in urban areas and moderate land occupation [36]. Beginning in the 1980s, however, a model of massive growth was promoted [37], linked essentially to the development of tourism and construction. On average, residentialist towns include a residential area growth of approximately 350% in their master plan, although the behaviour can be uneven. As shown in Table 3, these increases were in some cases tenfold (Poble Nou de Benitatxell, Castell de Guadalest and Adsubia), and in pre-existing urban areas, they multiplied by up to 20 (Llíber). By contrast, the projected growth was adjusted in very few cases to more moderate or even regressive demographic trends.

In these environments, characterised by a high elderly population, the adoption of friendly measures has also been uneven. In general, accessibility improved throughout the built-up area and there was a greater provision of services. A network of local public facilities and green spaces was also consolidated, which improved living conditions in some towns (Table 4).

Nevertheless, beyond the improvements in the structure and function of urban areas, socio-spatial integration can vary widely in each case. In this respect, many towns ultimately develop differentiated dual societies within their own municipal boundaries. This has had a number of different effects. On the one hand, the residentialist areas are private in nature, are often clearly differentiated from the traditional urban core and incorporate services that frequently only target this population. On the other hand, there is a consumerist and productive duality, since the functions of residentialist spaces appear to be linked more to leisure activities than to productive ones, setting the tone for the local population’s daily life. Finally, culturally speaking, foreigners usually limit their participation in the activities deemed essential by the native population (e.g., traditional local festivals) that new residents may find difficult to adopt. For this reason, in some towns, the new residents’ daily life ultimately revolves around their own national community (such as the Norwegians living in the area around Alfaz del Pi and La Nucia), thus weakening the links between and social integration of the native and foreign populations.

## 4. Discussion

Based on the results obtained, the reasons for the residentialist phenomenon appear to be the same as those highlighted in the 1970s, though with some small divergences [16]. The mild climate, especially in the non-summer months, is the main reason given by almost all groups and in almost all areas [21,26]. Price differences are still commonly referred to, particularly housing prices, although these disparities have narrowed over time because of Spain’s development and its integration into the joint EU economic area. The third major reason is the lifestyles in small towns and coastal areas, which are quieter and more informal [2,11].

The factors are generally closely linked to the senior population’s needs: favourable weather conditions for migrants attracted more by residential than work factors; affordable prices for people who often depend mainly on a pension; and a quiet life that is much less stressful than in big cities. As for the disadvantages, language is usually the main reason, as the vast majority do not speak Spanish. This prevents full participation in community life and at the same time encourages or forces the formation of distinct national communities. In addition, leisure time and quality of life in these communities is linked to a wide range of differentiating factors: sociodemographic, residential and health reasons, resources or personal contacts [38].

On the other hand, the settlement of retired migrants has appeared to increase the resources available at a destination. They have largely facilitated the flow of financial capital to the area, encouraging the purchase of homes by many other compatriots, given the existence of a welcoming society. Ultimately, however, few migrate definitively [18]. The retiree migrants have also fed the service economy in the area, in the face of all kinds of demands from new residents [39].

Generally, the negative impacts tend to be more noticeable in newly settled areas than in mature tourist destinations, where urban infrastructure is already well-established [40] and where they are considered to be part of a mode of economic production. The province of Alicante is no exception. Hoggart demonstrated the relationship between retired migrants and economic development of some areas in France [41]. Their arrival and settlement, the economic transformation that accompanies them and the income they represent for the host communities help to increase the resources available to local treasuries. In so doing, they also contribute to improved care for the local residents most in need of support, including the native elderly, who, for their part, do not perceive the settlement of these retired migrants as conflictual.

Yet, one may ask to what extent these environments can be considered friendly for the elderly population and aging process. It is somewhat difficult to address this point because the characterisations of age-friendly environments tend to focus on environments that are urban and on the native population [42,43,44]. In addition, the situation under study is unique and does not correspond to the demands of traditional rural towns either. Phillipson [45], when discussing good and optimal places to age, cites the advantages (medical and cultural advantages, as well as the varied leisure options) and threats (insecurity, vulnerability and major neighbourhood changes) of urban environments. In the case of residentialist towns, standard medical care is available. Insecurity is usually much lower than in large cities; cultural resources are fewer, but contact with natural spaces is more prevalent. Neighbourhood changes are not usually radical from a sociocultural point of view. Considering Plouffe’s requirements for friendly cities [46], some aspects of the province of Alicante clearly measure up, such as the availability of outdoor spaces, accommodation possibilities and security. Other requirements, such as social participation and inclusion, are met within the smaller, national collective itself, but less for the overall population in the area.

It is not easy to assess all these elements in the same way. For example, aging in one’s own home and community has been described as a substantial benefit [47]. Clearly, retired migrants are not in that situation. They have chosen a new home in which to reside after the working stage of their lives, often in the destination where they used to spend their annual holidays, and almost always with people who share a very similar national and sociocultural background. Active aging, as defined in studies on age-friendly environments [46,48], depends on multiple common factors: health, family and social networks or economic resources. Most of these communities tend to have access to all these resources and services, depending on their degree of personal autonomy. The large number of social clubs and contact centres that have arisen formally or spontaneously in many municipalities seem to attest to this.

The extent to which their arrival has also served to promote leisure opportunities and improve free time for the local elderly population in each area remains to be seen. The development of many Alicante municipalities—Torrevieja, Teulada-Moraira, Calpe, La Nucia, Alfaz del Pi, etc.— appears to show that the conversion to residentialism has had the following effects: (a) increasing not only the number of foreign retirees but also the total population, far beyond the retirees themselves; (b) encouraging the arrival of European workers with links to immigrants from their country of origin; (c) contributing to local wealth and fostering employment, favouring a shift to the tertiary sector that increases the variety and quality of the services on offer; and (d) increasing municipal budgets, permitting the development of welfare and cultural policies of all kinds that do not only target foreign groups. Clearly, the local elderly population has at its disposal a range of services, leisure opportunities, care resources and possibilities for personal development that would hardly have been possible without the transformative effect of residentialism.

However, some data show that residentialism is not a linear, growing and homogeneous process. For instance, between 2011 and 2021, the group shrank slightly for the reasons mentioned above (economic and political causes, as well as the health crisis). A certain spatial transfer also took place as the residential population decreased in size in the coastal areas and increased in interior rural areas. Moreover, some essential residential locations in 2001 have ceased to meet all the requirements to be categorised as residentialist, particularly the criterion of having an average age that is significantly higher than the average age in the province. Illustrations include La Nucia, the first example of intense residentialism, and Torrevieja, the largest Spanish city regarded as residentialist according to the established criteria, as well as in other tourist towns such as Calpe or Jávea.

The percentage of foreigners is not stable: it decreases when people return home or when deaths occur in their new homes and increases with new arrivals. In the case of residentialist groups—essentially of mature or advanced age—mortality rates are logically higher than in Spanish society as a whole. It is, moreover, common to return to one’s country after losing personal autonomy or following the death of a partner. A massive and steady influx of new migrants would be needed to merely maintain the size of the groups. However, it seems unlikely that the phenomenon will end any time soon [49].

Residentialism appears to be consolidated in the province of Alicante, as well as in other coastal and pre-coastal areas in southeast Spain. Indeed, in Almeria, the initial settlement around Mojácar is largely shifting to small inland municipalities such as Arboleas and Partaloa, where the foreign, largely residential, population now predominates [50].

Therefore, when examining the conversion of many residentialist municipalities into age-friendly environments, it is essential to bear in mind that while societies may well be aging, these are dual societies in which the situation for European foreigners is more complex. In their case, age friendliness may depend less on the homes where they live, financial insufficiency or the lack of green spaces. Although they may not suffer from poverty in the same way as many local seniors do, they often experience much more social exclusion, lacking the language skills to communicate with their neighbours, with fewer protective social networks around them and with personal autonomy issues that they must often face while cut off from their families. The adaptation required to create an age-friendly environment should perhaps rest less on urban features and more on reducing the duality often observed in daily coexistence in many residentialist areas. Building bridges of all kinds could well play an essential role.

## 5. Conclusions

This study confirmed that the settlement in recent decades of—mostly retired—foreigners in various municipalities in the province of Alicante has led to a significant population increase. This has been accompanied by a growth in local employment, available services and all sorts of opportunities. These municipalities present an average population age that is older than the total average population in the province. Nevertheless, once a residentialist society has become established, no strong population-aging tendency is seen to occur. On the contrary, age values tend to be closer to the provincial average. An obvious relationship with household size was found but not with the percentage of single people or income, nor even with a greater number of specialised services targeting the elderly population.

A number of aspects that interfered in the analysis must be considered. The model requires more complex data, as well as fieldwork in the areas under study. The data on the foreign resident population are affected by significant under-registration, which is much higher than that of the poorest migrant population—for whom registering and legalising their residence offers obvious advantages, such as access to health care, schools and social assistance. The actual number of people from northern Europe in the province of Alicante far exceeds the official census data. Moreover, incentives—such as participation in local elections—do not seem to have encouraged them to register their residence. For this reason, many municipalities face significant economic problems, since the population to which they must offer services—rubbish collection, security, social care, water and public lighting—is much larger than what is actually recorded. Although they bring substantial direct wealth (consumption taxes), indirect wealth (the consumption rates in these municipalities surpass that of their official population) and induced wealth (through increased activity in the area), their frequent choice to live outside the traditional urban centres also increases the costs of some municipal services.

In societies with a high percentage of elderly people, statistical underreporting and peripheral locations make it more difficult to meet the needs of a sensitive group and to collect the complementary resources required to shape age-friendly environments. All this involves multiple needs, relating both to social issues and the design of spaces and public services that promote healthy, active and integrated aging, at least within their own residential estates. This poses a significant challenge for age friendliness, perhaps because the requirements are very different from those of populations who age in the environment in which they have always lived.

Building and managing age-friendly environments may require conceptual and planning adaptations to integrate these differentiated groups. Maxims such as the advantages of aging in one’s own environment or processes such as basic spatial accessibility may need to be reviewed when intervening in the areas in which these groups live. It is also necessary to differentiate concepts such as poverty risk or social exclusion accurately when undertaking the difficult task of encouraging integration.

The arrival of the retired foreign population clearly increased the share of the elderly population in each affected municipality. This fact should lead to the promotion of public policies to improve the lives of the elderly overall, whether native or migrant. Their influx has also helped to develop the areas concerned, boosting the economy, providing resources to local society and, with it, improving the local infrastructure. They have thus facilitated the lives of those who, in those same municipalities, age in the place where they have always lived.

## Figures and Tables

**Table 1:** Evolution of the population of the province of Alicante, 2001–2021.

	Province of Alicante	Non-Residentialist	Residentialist	Semi-Residentialist
Num. of Municipalities	141	108 ^1^	18	9
Population (1 January 2001)	1,489,740	1,307,862	171,882	9542
% foreigners	8.9	4.5	41.9	22.0
% Europeans out of total foreigners	77.3	60.7	90.4	87.0
Median ages	39.3	38.5	44.9	45.0
Population (1 January 2011)	1,934,127	1,624,595	290,856	17,205
% foreigners	24.1	17.9	57.6	50.2
% Europeans out of total foreigners	71.0	30.7	86.3	92.3
Median ages	41.4	40.5	46.2	46.1
Population (1 January 2021)	1,881,762	1,624,488	240,403	15,568
% foreigners	19.8	15.8	45.6	38.9
% Europeans out of total foreigners	59.7	51.3	77.9	86.8
Median ages	43.4	43.0	46.2	47.0
% Population increase between 2001 and 2021	26.3	24.2	39.9	63.0
Increase in median ages between 2001 and 2021	4.1	4.5	1.3	2.4

^1^ Data from six municipalities, Benasau, Benillup, Famorca, Orxeta, Tollos and Vall de Alcalá, are not included because of a lack of comparative data (total or by age) for some years. None of these municipalities would be considered residentialist.

**Table 2:** Correlation between the foreign population of European origin and specific sociodemographic factors in the municipalities of the province of Alicante (between 2001 and 2021).

Factor A: Non-Spanish European Population in Each Municipality	Factor B: Other Sociodemographic Data	Municipalities in Which the Correlation Is Established	
		All the Municipalities in the Province	Only Municipalities with Fewer than 10,000 Inhabitants
% population with European nationality, 2001	Increase in the average age of the population, 2001–2021	0.28	0.36
% population with European nationality, 2001	Average household size, 2015	−0.16	−0.13
% population with European nationality, 2015	Average household size, 2019	−0.38	−0.36
% population with European nationality, 2019	% single-person households, 2015	−0.36	−0.34
% population with European nationality, 2015	% single-person households, 2019	0.05	−0.05
% population with European nationality, 2019	Average income per person, 2015	0.06	−0.03
% population with European nationality, 2016	Average income per person, 2019	−0.29	−0.33
% population with European nationality, 2020	Number of private opticians, orthopaedics and dental clinics, 2020	−0.36	−0.40
% population with European nationality, 2020	% population increase, 2001–2021	−0.15	−0.08
% population with European nationality, 2001	Increase in the average age of the population, 2001–2021	0.28	0.36

**Table 3:** Growth of residential areas according to city master plans (since 1981).

Municipality	City Master Plan Year	Consolidated Residential Area (Hectares) [A]	New Residential Development Area (Hectares) [B]	% Projected Increase in Residential Areas [B]/[A] × 100
Adsubia	1981	13	177	1383.4
Alcalalí	1992	71	58	82.6
Alfaz del Pi	1987	381	363	95.3
Algorfa	2002	50	212	424
Benidoleig	1985	21	93	441.4
Benigembla	1993	11	29	271.4
Benijófar	2005	112	103	92.1
Benimeli	1989	7	48	655.8
Benissa	1982	669	736	110
Bolulla	2009	5	2	47.6
Busot	1995	313	12	4
Calp	1998	980	260	26.5
Castell de Guadalest (el)	1993	5	71	1403.8
Daya Nueva	1995	27	27	101.0
Daya Vieja	2003	7	9	134.9
Fondó de les Neus (el)	1992	41	80	195.7
Hondón de los Frailes	1984	21	0	0
Jávea	1990	1930	1449	75.1
Llíber	1984	17	350	2065.7
Murla	1990	13	58	464.1
Nucia (la)	1988	354	479	135.2
Orba	1987	110	56	50.6
Parcent	2019	24	0	0
Poble Nou de Benitatxell (el)	1987	33	513	1545.4
Poblets (els)	1989	106	77	72.2
Ráfol d’Almúnia (el)	1988	56	95	169.7
Relleu	1997	38	8	20.7
Rojales	2000	432	605	140.1
Sagra	1996	17	13	77.2
San Fulgencio	1998	53	458	870.1
San Miguel de Salinas	1988	78	205	261.3
Sanet y Negrals	2012	16	39	247.8
Tárbena	1991	15	147	995.5
Teulada	2004	903	196	21.7
Tollos	1989	1	0	0
Tormos	2004	13	65	517.3
Torrevieja	2000	1246	886	71.2
Xaló	1994	162	154	94.9

**Table 4:** Area of sanitary services, public green spaces and local public facilities in residentialist municipalities (2021).

Municipality	Population in 2021	Sanitary Services Area (m^2^)	Public Green Spaces (m^2^)	Area of Local Public Facilities	
				m^2^	m^2^ per Inhabitant
Tollos	37	0	0	55,601	1503
Murla	549	397,223	6093	457,291	833
Llíber	883	0	110,168	654,162	741
Tárbena	642	0	248,951	457,906	713
Algorfa	3396	0	14,563	1,178,546	347
Relleu	1173	1125	126,470	358,866	306
Tormos	327	0	63,429	96,566	295
Benigembla	483	0	10,173	133,776	277
Poble Nou de Benitatxell (el)	4447	0	443,401	1,204,489	271
Ráfol d’Almúnia (el)	670	0	117,729	158,805	237
Benissa	11,462	2731	683,371	2,169,086	189
Parcent	983	451	14,383	160,391	163
San Miguel de Salinas	6553	0	308,477	1,006,517	154
Bolulla	426	11,479	8899	62,762	147
Busot	3207	3734	281,856	466,204	145
Alcalalí	1336	0	38,169	190,530	143
Sanet y Negrals	702	0	18,869	97,390	139
Benijófar	3323	5688	204,218	430,311	129
Fondó de les Neus (el)	2634	9321	79,401	332,270	126
Adsubia	609	254	13,990	71,076	117
Teulada	11,466	15,775	651,480	1,335,037	116
Benidoleig	1166	4587	55,713	133,877	115
Daya Nueva	1757	0	36,112	197,360	112
Sagra	427	116	6324	47,934	112
Xaló	2892	8768	77,817	300,752	104
Jávea	27,893	6044	1,829,532	2,644,158	95
Castell de Guadalest (el)	243	0	6066	20,451	84
Benimeli	415	265	20,554	32,547	78
Rojales	15,978	0	849,427	1,171,794	73
Orba	2233	0	4887	158,582	71
Calp	23,530	38,673	649,896	1,577,456	67
Poblets (els)	2721	4408	111,490	181,554	67
Torrevieja	82,842	290,947	1,660,281	3,861,375	47
Hondón de los Frailes	1299	0	1255	59,598	46
San Fulgencio	8447	0	169,449	365,372	43
Alfaz del Pi	20,495	15,000	257,007	829,167	40
Nucia (la)	18,108	0	69,758	687,436	38
Daya Vieja	707	0	15,661	22,648	32

## Data Availability

The data presented in this study are openly available in the National Statistics Institute of Spain at https://ine.es/, the Ministry of Health of the Generalitat Valenciana at https://dadesobertes.gva.es/ and the Ministry of Territorial Policy, Public Works and Mobility of the Generalitat Valenciana at https://politicaterritorial.gva.es (accessed on 25 December 2022).
